# Dengue Serotype Cross-Reactive, Anti-E Protein Antibodies Confound Specific Immune Memory for 1 Year after Infection

**DOI:** 10.3389/fimmu.2014.00388

**Published:** 2014-08-14

**Authors:** Ying Xiu Toh, Victor Gan, Thavamalar Balakrishnan, Roland Zuest, Michael Poidinger, Solomonraj Wilson, Ramapraba Appanna, Tun Linn Thein, Adrian Kheng-Yeow Ong, Lee Ching Ng, Yee Sin Leo, Katja Fink

**Affiliations:** ^1^Singapore Immunology Network (SIgN), Agency for Science, Technology and Research (A*STAR), Biopolis, Singapore; ^2^Communicable Disease Centre, Institute of Infectious Disease and Epidemiology, Tan Tock Seng Hospital, Singapore, Singapore; ^3^Environmental Health Institute, National Environment Agency, Singapore, Singapore; ^4^Yong Loo Lin School of Medicine, National University of Singapore, Singapore, Singapore; ^5^Lee Kong Chian School of Medicine, Nanyang Technological University, Singapore, Singapore

**Keywords:** Dengue, viral infection, antibodies, B cells, plasmablasts, longitudinal studies, cross-reactive, vaccines

## Abstract

Dengue virus has four serotypes and is endemic globally in tropical countries. Neither a specific treatment nor an approved vaccine is available, and correlates of protection are not established. The standard neutralization assay cannot differentiate between serotype-specific and serotype cross-reactive antibodies in patients early after infection, leading to an overestimation of the long-term serotype-specific protection of an antibody response. It is known that the cross-reactive response in patients is temporary but few studies have assessed kinetics and potential changes in serum antibody specificity over time. To better define the specificity of polyclonal antibodies during disease and after recovery, longitudinal samples from patients with primary or secondary DENV-2 infection were collected over a period of 1 year. We found that serotype cross-reactive antibodies peaked 3 weeks after infection and subsided within 1 year. Since secondary patients rapidly produced antibodies specific for the virus envelope (E) protein, an E-specific ELISA was superior compared to a virus particle-specific ELISA to identify patients with secondary infections. Dengue infection triggered a massive activation and mobilization of both naïve and memory B cells possibly from lymphoid organs into the blood, providing an explanation for the surge of circulating plasmablasts and the increase in cross-reactive E protein-specific antibodies.

## Introduction

An estimated 390 million people per year are infected with dengue, resulting in 100 million clinically apparent cases. There is no specific treatment available against dengue and clinical management of dengue patients is symptomatic. Fluid replacement and careful monitoring of patients can prevent fatalities in most cases. However, the efficacy of supportive treatment depends on available manpower and bed capacities in hospitals, which can become limiting especially during disease outbreaks (1). Therefore, efforts to develop a dengue vaccine have been ongoing for decades. The failure of previous attempts may be attributed to a combination of complications including insufficient attenuation of vaccine strains, limited knowledge about neutralizing epitopes, the lack of an immuno-competent animal model, and no defined correlate(s) of protection. The neutralization assay remains the gold standard to measure immunity to dengue, even though a correlation with protection has not been proven in humans (2). It appears difficult to standardize the assay due to its sensitivity to changes in temperature, incubation time, and pH (3, 4). Furthermore, a basic biological problem of the assay is that it cannot distinguish between cross-protective immunity and serotype-specific protective immunity. Epidemiological studies have shown that herd immunity to a prevalent serotype protects the population for several years against the serotype of infection but not against other serotypes (5, 6). On the other hand, an infection with one serotype confers temporary immunity to all four serotypes, showing that a cross-reactive component of the immune response elicits temporary tetravalent protection (7–10). On a population level, the cross-protective effect appeared to last for 1–3 years (11). On an individual level, experimental infection studies with humans suggest that cross-protection lasted as long as 9 months (12). Prospective cohort studies estimated that cross-protection lasted 1.6–2 years, based on the observation that re-infections during this time window were subclinical (8, 10). None of these studies, however, provide information about the serum antibody epitopes during acute disease and later during the period of cross-reactivity.

The coat of dengue virus particles consists of the structural proteins prM and E, which are assembled on a lipid bilayer derived from the host cell membrane (13). Ninety dimers of the E protein are arranged in a densely packed repetitive manner to form the virus particle and the E protein is the only protein exposed on mature virus particles (13). The E protein has three domains (EDI, EDII, EDIII) and antibodies against serotype-specific epitopes of EDIII have been described to be more potently neutralizing than Abs against EDI and EDII (14, 15). The pr component is cleaved during virus maturation, leaving behind the M protein, which spans the lipid bilayer and is hidden by E proteins. In mosquito and mammalian cell lines, PrM cleavage is incomplete and part of the released virus particle is partially or completely immature, resulting in the display of prM on released virus particle (16). Such immature virus particles are less infectious than mature virus particles. However, PrM-specific antibodies in patients may facilitate Fc-receptor-mediated infection via antibodies bound to prM on immature virus particles in a process called antibody-dependent enhancement (ADE) (17, 18). It has been shown that a significant fraction of antibodies in serum and antibodies isolated from immortalized memory B cells from healthy donors with a previous dengue infection bound to prM, whereas the remainder of the isolated antibodies bound to non-structural proteins and to the E protein (17, 19). In contrast to antibodies cloned from memory B cells, antibodies cloned from plasmablasts during the acute phase of a secondary infection were almost exclusively EDI or -II specific (20). There is evidence that the fusion loop of EDII in particular appears to constitute a major target of serum antibodies (21, 22). The fusion loop is essential for infection because it allows the virus to fuse with the host cell membrane in endosomes for the release of the viral RNA. Only a small percentage of the E protein-specific memory B cell-derived antibodies in humans were found to bind to EDIII (14, 19). Moreover, recent reports started to question the biological relevance of EDIII-binding neutralizing antibodies for protection (23, 24).

Apart from the study by Lai et al. (22), which addressed the binding of serum antibodies to two conserved amino acids in the fusion loop 3–18 months after infection, we are not aware of studies that characterized epitopes of dengue antibodies in longitudinal patient blood samples. Such data, however, can help to better understand the molecular basis of cross-reactivity during acute disease and in immune memory.

Here, we analyzed longitudinal plasma samples from patients with PCR-confirmed primary or secondary DENV-2 infection and tested the neutralizing capacity of plasma antibodies, their ability to bind to virus particles, to recombinant E protein, and to recombinant EDIII. We found that E protein-specific antibodies increased rapidly after infection. In contrast, EDIII-specific antibody responses varied substantially between patients and EDIII-specific titers generally dropped within 7 months after infection. The EDI and -II protein-specific antibody responses lasted for 1 year after infection and likely represented cross-protective immunity.

## Materials and Methods

### Patient samples

As part of the prospective Early Dengue (EDEN) infection and outcome study in Singapore (25), adult patients (age >21 years) presenting at community primary care polyclinics with acute onset fever (>38.5°C for less than 72 h) without rhinitis or clinically obvious alternative diagnoses, were included in the study. For the first cohort, a total of six whole blood samples were collected from each volunteer into EDTA-vacutainer tubes (Becton Dickinson) at recruitment (acute phase) and subsequently over a period of 1 year after disease onset (Table 1). For the second cohort, a total of two whole blood samples were collected into EDTA-vacutainer tubes during the acute phase of the disease and 3–4 months later (Table 2). Patients were diagnosed by DENV-specific RT-PCR (26) and by NS1 test (SD Bioline). The commercially available PanBio Indirect ELISA kit (Inverness Medical, Australia) was used to classify patients experiencing a secondary infection based on their IgG seropositivity in the sample collected within 72h after fever onset.

**Table 1:** **Cohort 1: longitudinal antibody analysis**.

Number of patients	Age; mean (range)	Sex	DENV-specific IgG <72 h after fever onset	Serotype of infection	Number of samples tested					
					<72 h	4–7 days	15–25 days	4 month	7 month	12 month
14	32.5 (15–56)	4 F, 11 M	−	DENV-2	13	13	13	14	13	11
12	40.9 (23–65)	4 F, 10 M	+	DENV-2	12	12	9	11	10	8

**Table 2:** **Cohort 2: cell phenotype analysis and PB correlations**.

Number of patients	Age; mean (range)	Sex	DENV-specific IgG <72 h after fever onset	Serotype of infection	V1: 4–7 days (mean 5.39 days)	V2: 4 months (mean 126 days)
15	36.47 (21–50)	3 F, 12 M	–	14 DENV-2, 1 DENV-1	15	15
13	35.77 (21–67)	1 F, 12 M	+	12 DENV-2, 1 DENV-1	13	9

### Ethics statement

This study was conducted according to the principles expressed in the Declaration of Helsinki. The research, which involved fever patients enrolled in the study was approved by the Domain Specific Review Board of Singapore’s National Healthcare Group (Domain E) and patients gave written informed consent (DSRB Refs. B/05/013, 2010/00227, and 2011/01770).

### ELISA

Maxisorp plates (Nunc) or half-area plates (Greiner) were used for all ELISAs. For the detection of virus particle-specific antibodies, ELISA plates were coated with PEG-precipitated and UV-inactivated DENV serotypes 1–4. For E protein-specific ELISA, the soluble part of E proteins of DENV1-4 with a His-tag were produced in S2 cells as described previously (27). For EDIII-specific ELISA, EDIII of DENV1-4 with a His-tag was produced in S2 cells, using the strategy described for Yellow Fever Virus in Ref. (28). Proteins were purified using Ni-beads. Half-area ELISA plates (Greiner) were coated at 75 ng/well. For all ELISAs, plates were blocked with PBS, 0.05% Tween 20 (PBST) and 3% skimmed milk. Sera and a standard containing pooled dengue-IgG positive plasma were diluted in blocking buffer on the coated plates for 1 h at RT before washing with PBST. Threefold serial dilutions from 1:200 to 1:48600 were tested. To standardize results between plates, a positive control was included on every plate and a ratio was calculated as follows: ratio of sample x = (OD_450(sample x)_ − OD450_(blank)_)/(OD_450(positive control)_ − OD450_(blank)_). The ratio of the positive control is therefore 1 for all ELISA graphs. For clarity, the ratio of only one serum dilution (1:600) per sample and time point is shown in all Figures. Pooled serum from dengue-naïve healthy donors was used as a negative control and included on all plates. Anti-human IgG-HRP (Sigma) was added at a concentration of 1:2000 and incubated for 1 h at RT. After washing, 3,3,5,5-tetramethylbenzidine HRP substrate solution (Sigma) was added. The color reaction was stopped with 1 M HCl.

### Neutralization assay

For the measurement of neutralization, heat-inactivated plasma samples, heat-inactivated pooled dengue-naïve plasma as a negative control and antibody 4G2 as a positive control were serially diluted in RPMI medium in 96-well plates (serum was diluted threefold over six wells starting with 1:200, mAb 4G2 starting concentration was 5 μg/ml) and a constant amount of virus (MOI 0.1–1) was added. The antibody-virus mixtures were incubated at 37°C for 30 min and then 50 μl of the mixtures were transferred to another 96-well plate containing 200,000 U937 cells (ATCC) stably transfected with human DC-SIGN (MOI 0.1-1) per well. After 2 h of infection, 150 μl RPMI medium containing 10% FCS was added. After incubation over night, the infected cells were fixed and stained intra-cellularly with 4G2-Alexa 647. The percentage of infected cells was quantified by flow-cytometry and data were analyzed with GraphPad Prism software for the calculation of the 50% neutralizing titer (NT50).

### Cell lines and virus strains

All viruses used were produced in C6/36 mosquito cells (ATCC). The following patient isolate strains were used: DENV1-05K2916 [EU081234 (29)], DENV2-TSV01 (30), DENV3-VN32/96 (EU482459), and DENV4-2641Y08 (isolated by Environmental Health Institute, Singapore). DENV3-VN32/96 was a gift from Dr. Cameron Simmons, Oxford University Clinical Research Unit, Viet Nam.

### Flow-cytometry analysis

White blood cells were isolated from 8 to 10 ml whole blood using CPT (BD) tubes containing Sodium Citrate as an anti-coagulant. Cells were counted and one million cells were stained directly for flow-cytometry analysis or all cells were frozen in RPMI medium containing 20% FCS and 10% DMSO. Frozen cells were thawed in batches for flow-cytometry analysis. Antibodies were purchased from Biolegend, except for anti-CXCR5-biotin (BD Pharmingen) and anti-CD14-ECD (Beckman Coulter). CXCR5-biotin was labeled with Streptavidin-APC-Cy7 (Biolegend). Cells were stained on ice for 30–60 min and washed with FACS buffer (PBS, 2% FCS, 5 mM EDTA, 0.1% sodium azide). Fixable blue live-dead marker (Invitrogen) was added freshly to the antibody mix and was incubated together with the surface marker antibodies. Cells were washed and fixed with 1% formalin for analysis.

### Statistical analysis

Data were initially analyzed with transMART (31). Data sets found to be significant were further analyzed with Prism Software (version 6 for Mac) and the statistical tests used were indicated in the figure legends. A *p* value of <0.05 was considered statistically significant.

## Results

### The acute antibody response is dominated by cross-reactive, E protein-specific antibodies, which circulate in the blood for less than 1 year

**Figure 1:**
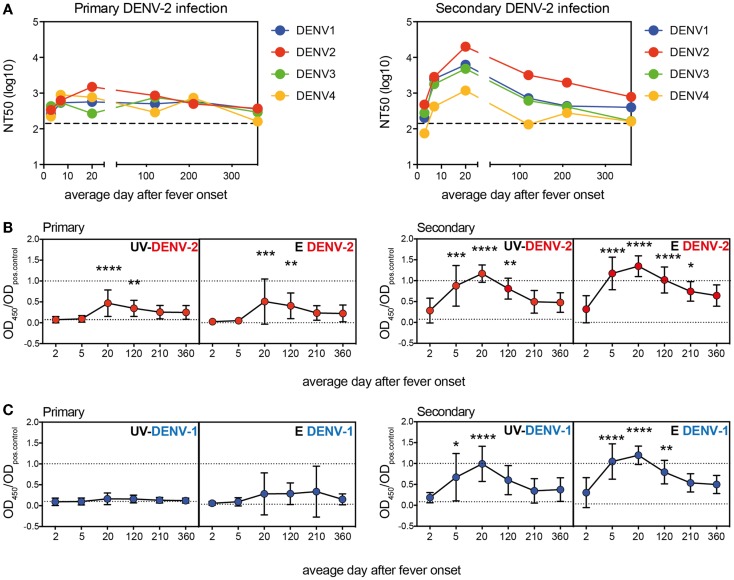
**More rapid generation of E protein monomer/dimer-specific Abs compared to virus particle-specific antibodies**. Plasma samples from patients with a primary or a secondary DENV-2 infection were analyzed at the indicated time points after onset of fever. **(A)** Neutralizing titers against all four DENV serotypes were measured. Dashed lines indicate the level of detection of the assay. Dots represent NT50 geometric means of nine donors per group. **(B,C)** ELISA to test binding of plasma antibodies to UV-treated, PEG-enriched DENV, or to recombinant E protein of DENV-2 **(B)** and DENV-1 **(C)**. A one-way ANOVA test was performed. *p < 0.05, **p < 0.01, ***p < 0.001, ****p < 0.0001 indicate titers that were significantly different from the titers measured within 72 h of fever onset (average day 2). Dashed lines indicate values for the positive and negative control, respectively.

The DENV-neutralizing activity of patient plasma is the average of binding capacities of a mixture of antibodies with different specificities. Antibodies binding to E protein epitopes and possibly also to prM epitopes can potentially be neutralizing (4, 32). With the aim to better define the specificity of polyclonal antibodies during disease and after recovery, longitudinal samples from 13 patients with primary DENV-2 infection and from 12 patients with secondary DENV-2 infection were collected over a period of 1 year, with the first blood sample being drawn within 72 h after fever onset (Table 1, Cohort 1). Neutralizing antibodies to all four DENV serotypes were quantified for nine primary and nine secondary patients with a flow-cytometry-based assay using U937-DC-SIGN cells. All longitudinal samples per patient were measured together in the same experiment. Since acute sera are highly neutralizing the lowest serum dilution used was 1:200 and the assay was not designed to accurately determine NT50 titers below this dilution. Whereas the response to DENV-2 dominated in both primary and secondary patients, cross-reactivity was observed particularly for the secondary patient group, and this cross-reactivity lasted for up to 1 year after the infection (Figure 1A). Cross-neutralizing activity of polyclonal antibodies is well described (33) and is thought to contribute to protection in individuals with one or multiple previous infections (9, 34), yet the epitopes of the cross-reactive polyclonal antibodies during the period of cross-protection are less well defined. We analyzed the specificity of DENV-binding polyclonal antibodies in plasma using ELISAs coated either with recombinant E protein or with PEG-precipitated, UV-inactivated virus particles (UV-DENV) (Figures 1B,C). As for the neutralization assay, all longitudinal samples per patient were analyzed together in the same experiment. An SDS-PAGE analysis of the recombinant DENV-2 E protein showed two bands, a smaller band for the monomer (40 kDa) and a less prominent band for the dimer (80 kDa) (Figure S1 in Supplementary Material). Recombinant E protein can spontaneously form dimers via the dimerization domain EDII (35) and both monomer- and dimer-specific antibodies are possibly detected in the E ELISA. In contrast to antibodies binding to recombinant E protein, antibodies binding to UV-DENV particles can be specific for E protein monomers, dimers, or for quaternary structures spanning more than one E dimer (36). We found that antibody titers to UV-DENV and to E protein both peaked between day 15 and 25 after onset of fever for the serotype of infection, DENV-2 (Figure 1B), and that antibodies cross-reacting with DENV-1 showed a similar kinetics (Figure 1C). Interestingly, titers against E protein of DENV-2 and DENV-1 increased more rapidly than titers against UV-DENV-2 and UV-DENV-1 in secondary patients.

These data showed that cross-reactive antibodies targeted at E protein increased during acute infection and dropped to almost baseline within 1 year. The different early kinetics between E- and UV-DENV-specific titers further suggested that a majority of B cells secreting antibodies during the acute infection were specific for the E protein monomer or dimer, and that less B cells seemed to be specific for complex viral epitopes, which would only be detected in the UV-DENV ELISA. Similar to the kinetics of the ELISA, both the specific and cross-reactive neutralizing response declined within 1 year.

### Pre-existing IgG titers are not indicative of the acute antibody response

**Figure 2:**
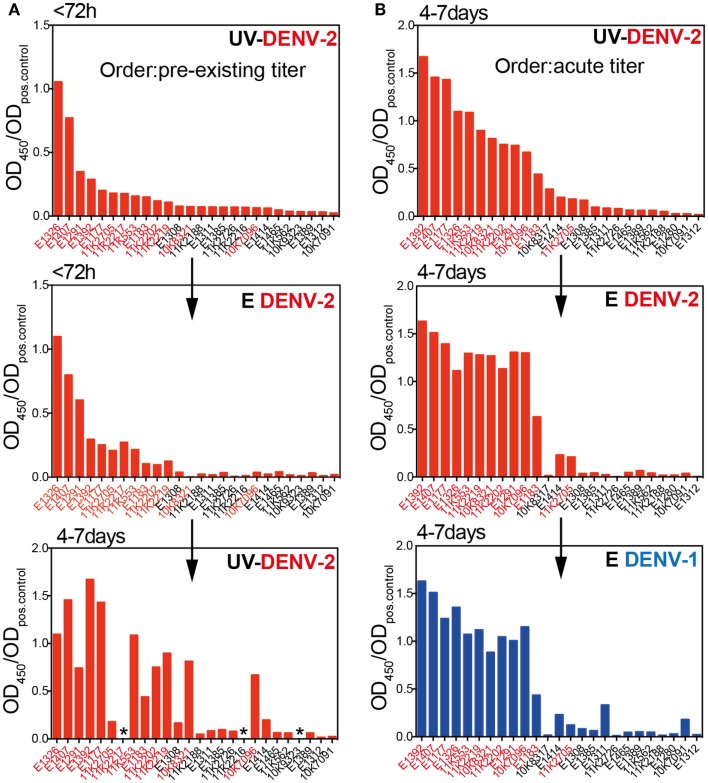
**Pre-existing dengue-specific IgG antibodies are not indicative of the anamnestic IgG response 4–7 days after fever onset**. **(A)** Plasma samples were sorted according to the UV-DENV-2 titer measured at <72 h and the values were compared with the E DENV-2 titers at the same time point and with UV-DENV-2 titer measured 4–7 days after onset of fever. **(B)** Plasma samples were sorted according to their UV-DENV-2 titer measured at 4–7 days after onset of fever and compared with E DENV-2 and E DENV-1 titers at the same time point. Patient IDs in red indicate secondary infections whereas patient IDs in black indicates primary infections. The classification primary versus secondary infection was made based on pre-existing IgG measured with the commercial indirect PanBio ELISA kit (see Materials and Methods) in samples collected <72 h after fever onset. *Indicates samples that were not available.

Patients were classified as dengue-naïve (primary infection) or dengue-immune (secondary infection) based on the pre-existing IgG titer measured in the <72 h sample using the commercially available PanBio indirect IgG ELISA kit (37). Data obtained with our in-house E protein ELISAs in Figure 1 showed that titers specific for E protein increased rapidly between the first (<72 h) and the second time point (4–7 days). This indicated that the early anamnestic response would be more accurate than pre-existing titers to distinguish dengue-naïve from DENV-immune patients and that the E protein ELISA could be more sensitive than the UV-DENV ELISA in distinguishing patients with primary or secondary infections. To test this hypothesis, we compared titers from the two early time points measured with both E- and UV-DENV ELISA. We first compared OD ratios measured in the UV-DENV ELISA <72 h after fever onset with E ELISA titers at the same time point and with UV-DENV ELISA titers measured 4–7 days after fever onset (Figure 2A). UV-DENV OD ratios measured <72 h after fever onset did not show a clear cut-off between primary and secondary patients (as classified based on PanBio kit) and the samples did not cluster at the 4–7 days time point when arranged in the same sequence (Figure 2A). In contrast, when the UV-DENV and E ELISA OD ratio was measured at 4–7 days (Figure 2B), 11 out of 12 secondary cases (patient ID on *x*-axis in red, based on PanBio kit) had significantly higher E ELISA OD ratios compared to primary cases (patient ID on *x*-axis in black, based on PanBio kit). The segregation into primary and secondary groups was evident for both DENV-2 and DENV-1 E protein ELISA, showing that day 4–7 antibodies were largely cross-reactive.

In summary, while E and UV ELISA titers were comparable at the <72 h time point, the E ELISA appeared to be more sensitive than the UV-DENV ELISA in distinguishing primary from secondary dengue patients when measured at defervescence (4–7 days after fever onset), in line with the finding that the majority of the anamnestic response seemed to be specific for E protein monomers and dimers.

### Serotype-specific E protein-, but not EDIII-specific antibodies remain elevated for at least 1 year after infection

**Figure 3:**
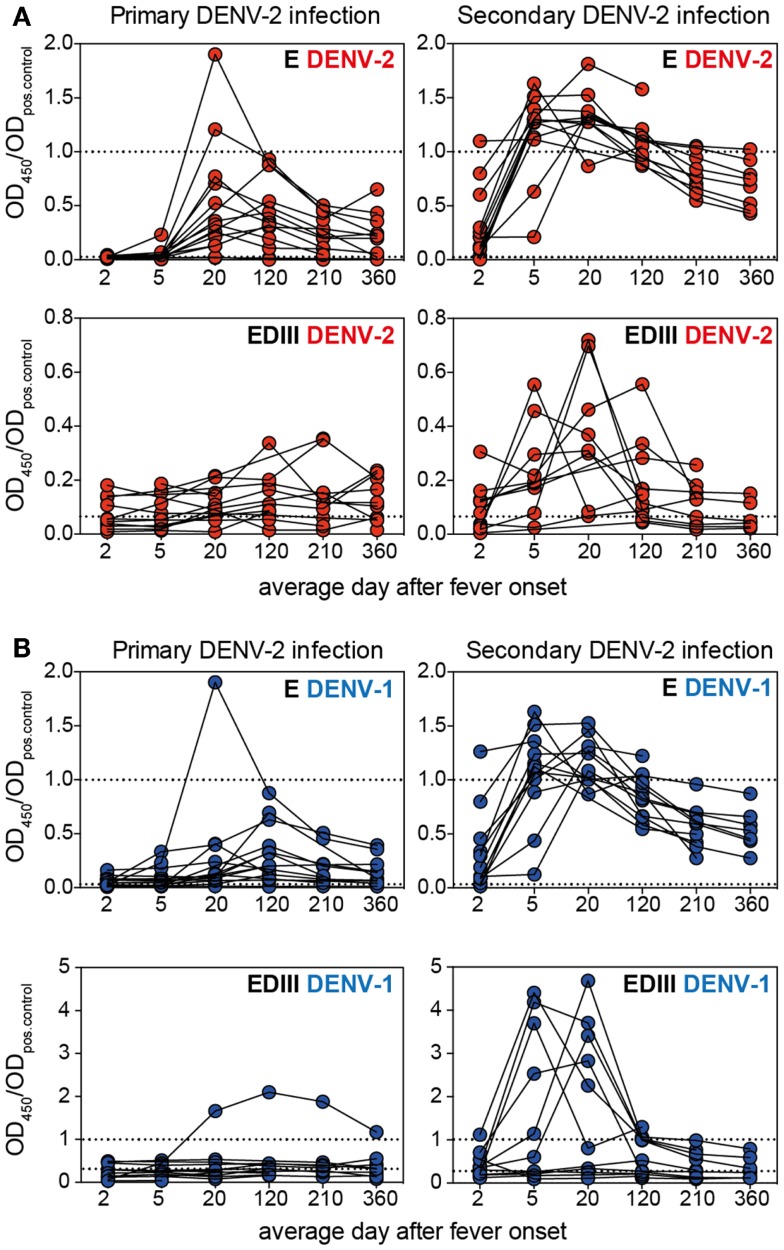
**The EDIII-specific antibody titers vary substantially between patients and are not long-lasting**. Plasma samples from patients with primary or secondary infection were analyzed for binding to E and EDIII protein and each patient’s antibody binding profile is shown individually by connected symbols. The mean values for the same data are shown in Figures 1B,C. **(A)** Individual curves for binding to DENV-2 E and EDIII protein. **(B)** Individual curves for binding to DENV-1 E and EDIII protein. The dashed lines indicate values for pooled neg. and positive control plasma, respectively. Mouse mAb 3H5 was used as a positive control for the DENV-2 EDIII ELISA because the pooled positive plasma did not show more binding to DENV-2 EDIII than the pooled negative plasma. The use of a different positive controls for DENV-1 and DENV-2 resulted in OD ratios of a different scale for the two EDIII ELISAs.

Several recent publications suggested that EDIII-specific antibodies, although often highly neutralizing and serotype-specific, are rare in the repertoire of dengue-immune individuals (14, 38). We assessed whether, despite their low relative abundance, EDIII-specific antibodies correlated with NT50 or E ELISA titers in our cohort. No convincing correlations between EDIII titers and E titers or between EDIII titers and NT50 were observed (data not shown). Surprisingly, neither primary nor secondary patients generated a strong long-lasting response against DENV-2 EDIII, in contrast to the robust DENV-2 E protein-specific response observed. Patients with a transient DENV-2 EDIII-specific response were found mostly in the secondary infection group (Figure 3A). Similarly, a transient DENV-1 EDIII-specific response was observed in the secondary patient group, and the samples that were positive for DENV-1 EDIII were also positive for DENV-2 EDIII (Figure 3B), suggesting that mostly conserved epitopes in EDIII accounted for the titers. Interestingly, one primary patient developed high titers of DENV-1 EDIII-specific antibodies after DENV-2 infection. The response was highly specific for DENV-1, with no detectable binding to DENV-2 EDIII. Even though a more sensitive Taqman qRT-PCR (39) was performed in addition to the SYBR-green based serotype-specific qPCR we could only detect DENV-2 – and no DENV-1viral RNA in the plasma of this patient. A potential alternative explanation for the DENV-1 specific response could be a molecular mimicry of EDIII with a self-or non-self antigen.

In summary, EDIII-specific serum antibodies were mostly cross-reactive and did not correlate with E protein-specific antibody titers. The EDIII-specific response seemed to vary substantially between patients. The reason for this is unclear, but it is an important point to consider when studying small numbers of patient samples.

### Cross-reactive neutralizing antibodies correlate with acute E protein-specific antibodies but not with E protein-specific antibodies from the immune memory phase

**Figure 4:**
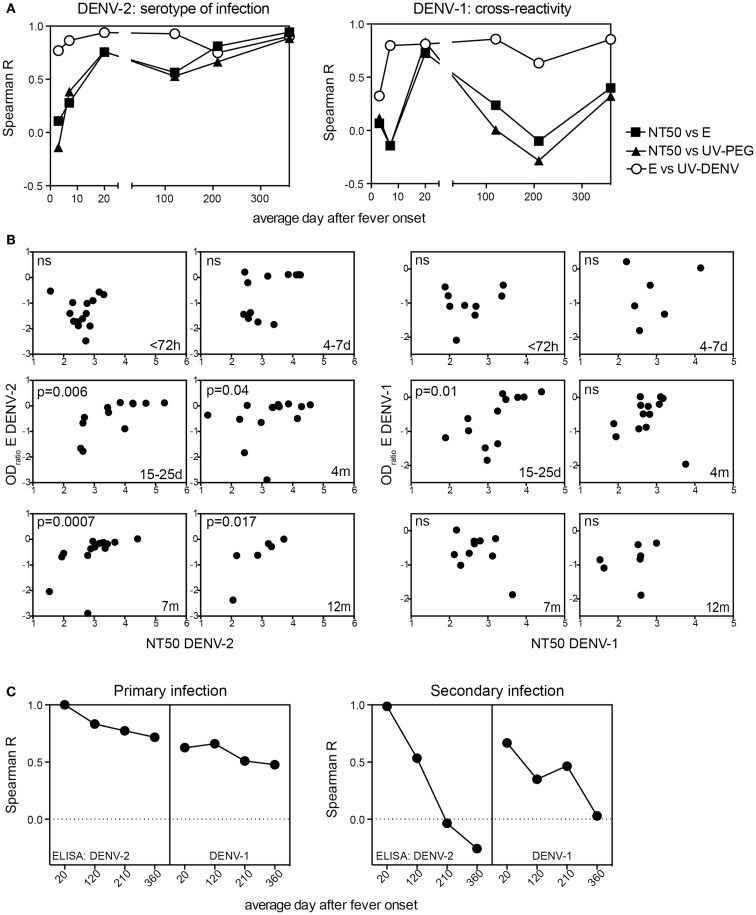
**Long-term correlation between E protein binding and NT50 for the serotype of infection**. Correlations of E protein- and UV-DENV-ELISA titers with NT50 for plasma from primary and secondary patients were calculated individually for the indicated time points of disease. **(A)** Spearman R for the correlations between NT50 and ELISA titers are shown for each time point. **(B)** Graphs illustrating the data points used for the calculations of Spearman R shown in **(A)**. Correlations for DENV-2 specific antibodies are shown in the graphs on the left and correlations for DENV-1 specific antibodies are shown in the graphs on the right. **(C)** Correlations between the highly cross-reactive E protein ELISA titer measured at day 15–25 for DENV-2 and the DENV-2 and DENV-1 E protein ELISA titers measured at time points indicated in the *x* axis were calculated. The correlations were calculated separately for primary and for secondary patients.

Since both the E protein-specific titers and NT50 started to increase as early as 4–7 days after infection (Figure 1) we tested whether there was a correlation between these two readouts, and whether the correlation was consistent over time (Figure 4). For DENV-2, the serotype of infection, a significant correlation between NT50 and E ELISA titers was first seen 15–25 days after fever onset and this correlation was consistent for at least 1 year. In contrast, the correlation between DENV-1 NT50 and DENV-1 E ELISA was only significant 15–25 days after fever onset and not for the later time points (Figure 4A). Only NT50 values calculated from a neutralization curve with a good fit (*R*^2^ value) were included in the analysis. Day 4–7 time point samples were difficult to analyze in the DENV-1 neutralization assay, even when starting with a lower serum dilution. As a consequence of this the correlation for DENV-1 at 4–7 days did not contain enough data points for interpretation and was only included for the sake of completeness (Figure 4B). However, we could conclude that during early convalescence (15–25 days), E protein-specific DENV-1/2 cross-neutralizing antibodies were likely responsible for the significant correlation between NT50 and ELISA for both homologous and heterologous serotypes. At late time points DENV-1 cross-neutralizing activity and DENV-1 “cross-binding” ELISA titers appeared to decline with different kinetics, resulting in a low correlation between DENV-1 NT50 and DENV-1 ELISA. The correlation in Figures 4A,B included both primary and secondary patients because the number of samples was too small to analyze the two groups separately. To address different correlation outcomes for primary and secondary patient due to different levels of cross-reactivity we compared E ELISA titers measured at the most cross-reactive time point (15–25 days) with E ELISA titers measured at later time points (Figure 4C). Primary patients showed a consistently high correlation between DENV-2 E protein-specific antibodies measured at day 15–25 and DENV-2 E protein-specific antibodies measured at later time points, and the correlation between DENV-2 E protein-specific antibodies measured at day 15–25 and DENV-1 E protein-specific antibodies at later time points was only slightly less consistent. In contrast, secondary patients’ DENV-2 E-specific antibodies measured at day 15–25 did not correlate with DENV-2 and DENV-1 E-specific antibodies measured at late time points (Figure 4C). We concluded that while the cross-reactivity correlations were markedly different between primary and secondary patients the readouts for DENV-2 and DENV-1 ELISAs were similar, suggesting that the different correlations associated with DENV-2 and DENV-1 reactivity observed in Figure 4A were a consequence of changes in the NT50.

In summary, the anti-E protein antibody response in primary patients measured 20 days to 4 months after infection correlated with the response measured 1 year after infection. In contrast, the response in secondary patients measured 20 days after infection was not predictive of the outcome measured 1 year later, suggesting that the E protein-specific cross-reactive antibody response waned within 1 year and that the response became more serotype-specific within this time frame. Correlations of NT50 and ELISA data might be useful to assess serotype-specific and cross-protective immunity as early as 4 months after infection (Figure 4A). However, since the patient cohort analyzed here only included DENV-2 infected patients, it will be important to test whether a similar correlation can be observed in a cohort with different serotypes of previous and new infection.

### Both naïve and memory B cells are activated after DENV infection and contribute to the cross-reactive antibody response

**Figure 5:**
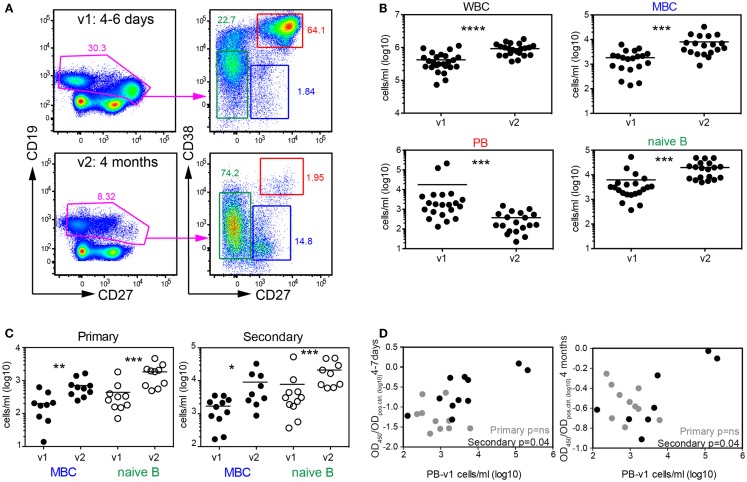
**Systemic mobilization of naïve and memory B cell both during primary and secondary infection**. **(A)** PBMCs from patients in the second cohort (Table 2) collected 4–7 days and 4 months after fever onset were stained for flow-cytometry analysis. Cells in the graphs were gated on live and CD14-negative cells. CD38 and CD27 expression on CD19^+/low^ cells was then used to differentiate between memory B cells (MBC), naïve B cells, and plasmablasts (PB). Graphs from one representative patient are shown. **(B)** Quantification of the cell subsets illustrated in **(A)**. **(C)** Data in **(B)** were separated into patient groups experiencing a primary or secondary infection. **(D)** Correlation between PB numbers detected at v1 and UV-DENV ELISA OD ratio (OD_450_/OD_pos.control_) measured at v1 and v2. **(B–D)** Each dot represents one patient; *p < 0.05, **p < 0.01, ***p < 0.001, ****p < 0.0001 (Wilcoxon matched-pairs signed rank test).

We next tested, which B cells could account for the rapid increase in DENV-specific antibody titers. Since primary patients do not have memory B cells specific for dengue we assumed that more naive B cells would be activated in primary patients compared to secondary patients, and that the higher antibody titers in secondary patients would be contributed mostly by memory B cells since memory B cells differentiate into plasmablasts more efficiently than naïve B cells (40). We analyzed blood B cells from patients during acute disease (between day 4–7 after fever onset, visit 1) and 3–4 months later (visit 2) by flow-cytometry and measured changes in absolute numbers for naïve B cells, memory B cells and plasmablasts (Table 2, Cohort 2 and Figure 5A). As reported previously by us and by others, a significantly higher number of plasmablasts was detected during visit 1 compared to visit 2, and the number of white blood cells in dengue cases was significantly lower during visit 1 compared to visit 2 (41, 42) (Figure 5B). In the acute phase we also observed a significant drop in memory B cell numbers, one hypothesis for which is that a majority of memory B cells had differentiated into plasmablasts (Figures 5A,B). Interestingly, decreased memory B cell numbers during acute disease were observed for both primary and secondary patients (Figure 5C), suggesting that a large proportion of the activated memory B cells were not DENV-specific. To address whether re-activated memory B cells during secondary infection accounted for long-lasting DENV-specific titers, we compared plasmablast numbers during acute disease with titers measured 4 month after infection (Figure 5D). While a weak correlation existed between plasmablast numbers and DENV-specific titers for secondary patients during acute disease and 4 months after infection, no such correlation was observed for primary cases. This finding indicated that primary infection plasmablasts were activated as “bystanders” and that the number of plasmablasts did not correlate with DENV-specific titers at a later time point.

**Figure 6:**
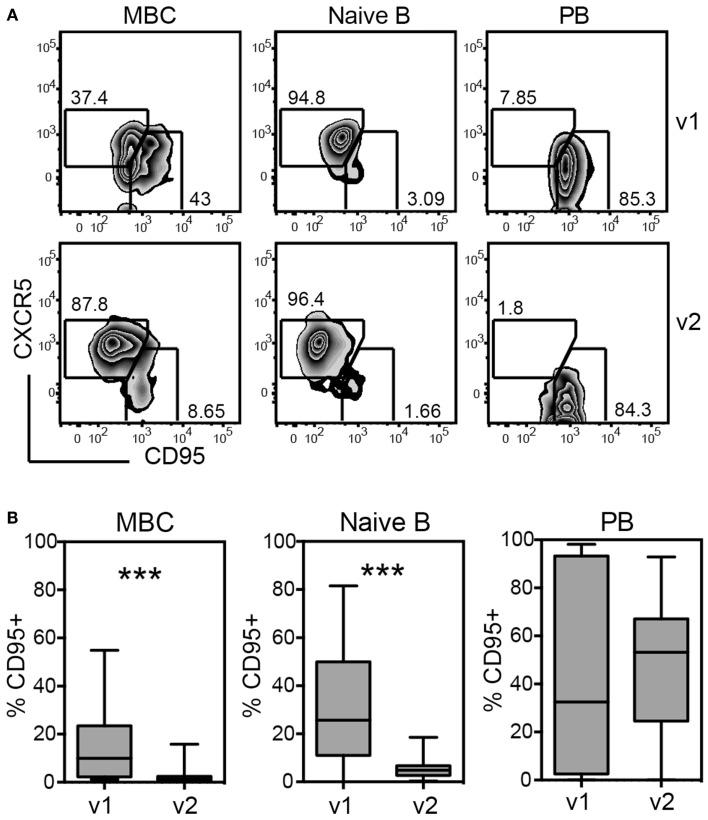
**Up-regulation of Fas-ligand (CD95) on memory and naïve B cells during acute disease indicates differentiation to plasmablasts (PB) and impending cell death**. PMBCs shown in Figure 5 were stained for CXCR5 and CD95 and the expression of these markers was analyzed on MBCs, naïve B cells, and PBs, including all 28 patients of cohort 2 regardless of primary or secondary infection. **(A)** Gating of CXCR5 and CD95-expressing cells during v1 and v2 for one representative patient. **(B)** Quantification of the percentages of CD95^+^ cells gated as illustrated in **(A)**. ***p < 0.001 (Wilcoxon matched-pairs signed rank test)

Fas-receptor (CD95) was significantly up-regulated on both naïve and memory B cells at visit 1 compared to visit 2, exposing the cells to Fas-mediated killing (Figures 6A,B). Loss of CXCR5 and up-regulation of CD95 occurred in parallel, suggesting that the majority of activated B cells did not home back to lymphoid tissues and were prone to die (Figure 6A).

Taken together, dengue infection seemed to systemically activate both naïve and memory B cells based on the increase in CD95-expressing cells and based on the reduction in total cell numbers for both populations.

## Discussion

Longitudinal analysis of antibody specificities and flow-cytometry-based analysis of B cell activation and mobilization revealed the following key characteristics of the DENV-specific humoral response: (i) the anamnestic response in secondary patients was highly dominated by cross-reactive E protein monomer- or dimer-specific antibodies and this response clearly differentiated secondary from primary patients. (ii) The E protein-specific, DENV serotype cross-reactive antibody response subsided within 1 year after infection.

Titers to EDIII remained low in primary patients and were variable in secondary patients throughout the time period assessed. In contrast, B cells specific for EDI/II seemed to be activated efficiently in both primary and secondary patients (Figure 3). Furthermore, the increase in UV-DENV-specific titers was less significant compared to the increase in EDI/II-specific titers during acute disease in secondary patients (Figure 1). Two studies recently described highly neutralizing antibodies with footprints spanning the EDI-EDII hinge region and EDIII of two adjacent E proteins on virus particles (36, 43). Additionally, the EDI/II hinge-region was found to elicit serotype-specific immune memory: a “serotype swop” of hinge region amino acids was sufficient to abrogate neutralizing activity by up to 80% and the neutralizing activity of sera from animals immunized with “hinge-region-chimeric” viruses was specific to the serotype of the hinge region (44). While we found that complex epitope-specific antibodies that only bound to virus particles seemed to be less abundant than E protein monomer/dimer-specific antibodies during the acute response, cross-reactive hinge region-specific antibodies binding to E protein monomer/dimers could be responsible for the increasingly serotype-specific response at later time points (Figure 4).

Memory B cells do not actively secrete antibodies and, unlike long-lived plasma cells, do not contribute to the serum antibody repertoire after recovery. However, memory B cells can be efficiently activated after a re-exposure to dengue virus. The data presented here suggest that memory B cells specific for conserved regions of EDI/II were activated abundantly, whereas this did not seem to occur for B cells specific for conserved regions of EDIII. It will be important for vaccine design to understand the mechanisms that drive such selective memory B cell re-activation.

During the analysis, we realized that it can be difficult to define true primary, DENV-naïve patients in a dengue endemic region. Pre-existing antibodies did not seem to be a good indicator, particularly in cases where the infection occurred a long time ago and where circulating DENV-specific antibodies may have waned over many years. Despite the absence of circulating antibodies, DENV-specific memory cells can still remain in the lymphatic organs and can be re-activated and expanded after infection, resulting in a rapid and high production of antibodies. Since secondary DENV infection is associated with a higher risk of developing a severe disease, an unambiguous test that is able to distinguish primary from secondary infection could be useful for clinicians to assess a patient based on a combination of warning signs of severe disease (2) and pre-existing immunity. Furthermore, a clear distinction between primary and secondary infection is important for dengue vaccine trials, since safety may have to be addressed separately in dengue-immune and dengue-naïve individuals. Drug trials may also benefit from an unambiguous distinction between primary and secondary patients since the kinetics and magnitude of viremia and NS1-positivity, which are used as readouts for efficacy, may differ between the two patient groups (45).

We suggest that E protein-specific antibodies measured at day 4–7 after fever onset are a sensitive biomarker to distinguish primary from secondary patients.

Besides this, cross-reactive antibodies produced during acute infection are relevant for protection. Human challenge experiments and epidemiological studies have shown that serotype cross-reactive responses contribute to protection (9, 12, 34). However, the cross-protection is temporary and it is important to understand at which time point the response becomes serotype-specific. Based on our results and results from other studies (33), the recommendation for vaccine testing would be to measure titers only 1 year after immunization to assess serotype-specific responses. Alternatively, the combined analysis of NT50 and E ELISA (Figure 4A) could potentially be useful to define serotype-specific immunity as early as 4 months after infection (Figure 4A). It would be interesting to test this correlation in other cohorts with different serotypes of infection.

The reason for the massive activation of B cells during acute dengue infection that seems to account for the cross-reactive response remains unclear. Specific activation of B cells by virus particles binding to the B cell receptor and unspecific activation via cytokines possibly act together (46). Recent evidence suggests that cytokines produced by CD14^+^CD16^+^ monocytes contribute to plasmablast formation in dengue patients (47). We show that both naïve and memory B cells were activated in primary and secondary patients based on CD95 expression, pointing to a substantial unspecific activation of B cells. It is remarkable that, despite the apparent unspecific B cell activation, a large proportion of plasmablast-derived antibodies in secondary patients bind to DENV (7, 20, 42, 48).

Previous studies defined the fusion loop of EDII as a major antibody target and reported that concentrations of antibodies binding to fusion loop amino acids W101 and F108 remained constant over 18 months after infection (22). The reason for the high immunogenicity of the fusion loop remains unclear. We detected decreasing E protein-specific titers over 12 months, which suggests that the fusion loop is only one of several epitopes in the E protein that accounts for cross-reactivity.

Plasma samples collected during acute secondary infection bound efficiently to recombinant E protein and less to virus particles. In contrast, steady state plasma samples collected <72 h or >7 months after fever onset contained antibodies that bound equally well to recombinant E protein and virus particles, therefore, likely representing a more diverse pool of antibodies. The results from this study suggest that EDII-specific cross-reactive antibodies were generated during the acute response and were mostly derived from re-activated memory B cells, whereas antibodies found in the plasma during long-term memory seemed to be more diverse. These findings can help to better understand differences in antibody specificities between the acute response and immune memory after dengue infection.

## Conflict of Interest Statement

The authors declare that the research was conducted in the absence of any commercial or financial relationships that could be construed as a potential conflict of interest.

## Supplementary Material

The Supplementary Material for this article can be found online at http://www.frontiersin.org/Journal/10.3389/fimmu.2014.00388/abstract
