# Supplementary material for: Dengue Serotype Cross-Reactive, Anti-E Protein Antibodies Confound Specific Immune Memory for 1 Year after Infection
Source: Front Immunol. 2014 Aug 14;5:388. doi: 10.3389/fimmu.2014.00388 (PMC4132268; doi:10.3389/fimmu.2014.00388)
Supplement: Supplementary file 1 [file Data_Sheet1.PDF]

Supplementary Figure 1

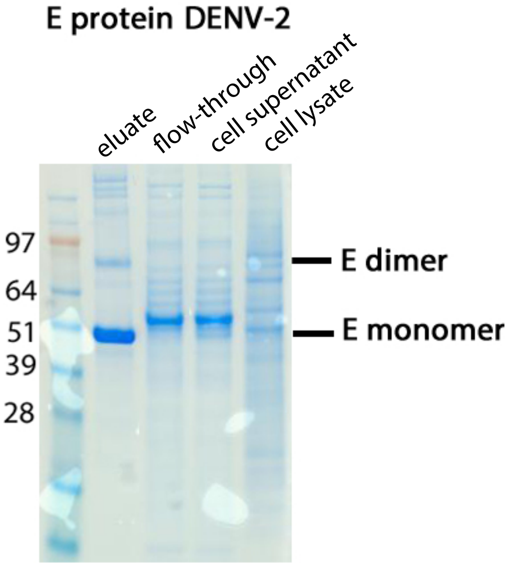

Eluate and flow-through from the purification of E protein using Ni-beads, together with cell supertnatant and cell lysate from E-protein producing S2 cells were analyzed by SDS-Page to test for correct size of E protein monomer and dimer and to test for the purity of the eluted protein.
